# Pharmacovigilance study of spinal epidural hematoma reports associated with direct oral anticoagulants and warfarin

**DOI:** 10.1007/s00701-026-06860-0

**Published:** 2026-04-30

**Authors:** Mokshal Porwal, Nassim Stegamat, Vinit Reddy, Stephen Jaffee, Dallas Kramer, Coby Cunningham, Alexander Yu

**Affiliations:** 1 Department of Neurosurgery, Allegheny Health Network, 320 E North Avenue, Pittsburgh, PA 15212 USA; 2 Department of Neurosurgery, Temple University, Philadelphia, PA USA; 3 Drexel University, Philadelphia, PA USA

**Keywords:** Epidural hematoma, Spinal, Anti-coagulation, Apixaban, Rivaroxaban, Warfarin

## Abstract

**Background:**

Spontaneous spinal epidural hematoma (SEH) is a rare but potentially catastrophic condition that may be worsened by anticoagulation. We evaluated reporting patterns and signal strength of SEH associated with warfarin and DOACs using pharmacovigilance database analysis.

**Methods:**

A retrospective disproportionality analysis was conducted using the FDA Adverse Event Reporting System (FAERS) from Q4/2003 to Q2/2025. Reports of SEH associated with warfarin, rivaroxaban, apixaban, and dabigatran were analyzed. Signal detection employed Reporting Odds Ratio (ROR), Proportional Reporting Ratio (PRR), and chi-squared analysis with Yates' correction. A signal was significant if n > 3, χ2 > 4, and PRR > 2.

**Results:**

A total of 143 SEH reports were identified. Warfarin accounted for 83 cases, rivaroxaban 28, apixaban 25, and dabigatran 7. All demonstrated significant disproportionality signals. Warfarin showed the strongest signal (ROR 29.2, 95% CI 22.9–37.1), followed by rivaroxaban (ROR 8.09), dabigatran (ROR 5.75), and apixaban (ROR 4.78). Warfarin patients were younger (mean 62 years) than DOAC users (75–76 years). Mortality occurred in 9 warfarin and 8 rivaroxaban cases. Atrial fibrillation was the most frequent indication for anti-coagulation.

**Conclusions:**

This pharmacovigilance study identified disproportionate reporting signals for spinal epidural hematoma across warfarin and DOACs, with stronger signals observed for warfarin and rivaroxaban. Given the inherent limitations of spontaneous reporting databases, these findings should be interpreted as hypothesis-generating rather than causal. Further controlled studies are needed to determine true incidence and comparative risk.

**Supplementary Information:**

The online version contains supplementary material available at 10.1007/s00701-026-06860-0.

## Introduction

Anticoagulation therapyis essential for preventing thromboembolic complications in conditions such as atrial fibrillation, venous thromboembolism, mechanical heart valves, and is used perioperatively in surgical procedures. While anticoagulants effectively reduce thrombotic risk, their mechanism of action inherently increases the risk of bleeding complications, ranging from minor bruising to life-threatening hemorrhage. It is vital to compare these agents with each other as they comprise diverse pharmacological classes with distinct mechanisms of action. Warfarin, a vitamin K antagonist, works to inhibit vitamin K epoxide reductase inhibiting the proper reduction of vitamin K and thereby preventing the subsequent activation of important coagulation factors. Direct oral anticoagulants (DOACs) targeting specific coagulation factors at different stages of the coagulation cascade. Each drug has different targets within the extrinsic or intrinsic coagulation cascade and therefore each carry potentially different bleeding risk profiles [10–14]. A spinal epidural hematoma (SEH) is a potentially surgical condition that occurs when there is a buildup of blood in the epidural space of the spinal canal. This can occur spontaneously or in a post-operative setting and can cause spinal cord compression. Due to the impending neurologic sequalae including paresis and death, surgical evacuation is often pursued to prevent neurological damage to the spinal cord [48, 68].

The connection between SEH and patients’ anticoagulant use is well known and understood as the mechanism is pathophysiological and there exists a dearth of literature regarding patients experiencing SEH while on anticoagulants [4, 7, 28, 30, 31, 34–36, 41, 52, 59, 61, 68]. Additionally, the pharmacokinetics of each anticoagulant is well understood and heterogeneous between individual medications. Despite all this understanding, safety data within the anticoagulant drug class remains limited, and the relative risk associated with DOACs compared to warfarin has not been systematically characterized in reference to spontaneous SEH. These differences need to be further elucidated in order to guide prevention of spontaneous SEH. It is with this understanding that we conducted a pharmacovigilance analysis to evaluate the reporting patterns and signal strength of SEH associated with warfarin and individual DOACs using the large Adverse Event Reporting System (FAERS) database.

## Materials and methods

### Literature search

A literature search for case series and case reports regarding spinal epidural hematomas associated with anti-coagulation use was conducted on PubMed using keywords such as: spinal epidural hematoma, anti-coagulation, warfarin, apixaban, betrixaban, dabigatran, edoxaban, and rivaroxaban. From our search, 119 articles were initially identified. Articles were selected using inclusion criteria such as: spinal epidural hematoma location and anti-coagulation use. Exclusion criteria included: iatrogenic sources of Epidural hematoma (steroid injection, epidural anesthesia), anti-platelet use only, hematomas in other spinal canal compartments (subdural, subarachnoid, intramedullary) (Table 1).

**Table 1:** Comprehensive review of literature for spinal epidural hematomas associated with anticoagulation

Study/Author	N	Spinal Lesion Location	Post Surgery vs Spontaneous	Age/Gender	Anti-Coagulation	Anti-Coagulation Indication	Requiring Surgical Evacuation	Increase in Morbidity vs Mortality
Barkas et al [4]	1	C2-C4	Spontaneous	72/M	Rivaroxaban	UNK	Y	Morbidity (LUE paraesis)
Krupa et al [28]	1	L1-L2	Spontaneous	84/F	Apixaban	Atrial Fibrillation	Y	Morbidity (4/5 in all muscle groups)
Li et al [30]	1	T5-T8, T12-L2	Spontaneous	80/F	Warfarin	Atrial Fibrillation	Y	Morbidity (significantly decreased strength, sensation + incontinence)
Limardo et al [31]	1	T5-T10	Spontaneous	81/F	Rivaroxaban + ASA	Unspecified Arrhythmia	Y	Morbidity (paraplegia)
Mathais et al [34]	1	C4-T5	Spontaneous	78/M	Dabigatran	Atrial Fibrillation	Y	None
Mezzacappa et al [35]	2	C2-T3	Spontaneous	73/F	Apixaban	Protein C deficiency	Y	Morbidity (R triceps 4/5)
		C2-T1	Spontaneous	84/M	Apixaban	Atrial Fibrillation	Y	Morbidity (LE Paraplegia, UE Paresis)
Montalbetti et al [36]	4	T10-T11	Spontaneous	71/F	Rivaroxaban	Atrial Fibrillation	Y	Morbidity (LE paraplegia + anesthesia)
		C3-T1	Spontaneous	80/F	Apixaban	Atrial Fibrillation	Y	Morbidity (L sided hypoasthenia)
		T3-T6	Spontaneous	81/F	Dabigatran	Atrial Fibrillation	Y	None
		L2-L3	Spontaneous	79/M	Apixaban	Atrial Fibrillation	Y	None
Pankongngam et al [41]	1	T8-L1	Spontaneous	65/F	Warfarin	Mechanical Valve Replacement	Y	UNK
Schoenmaekers et al [52]	1	C2-T5	Spontaneous	61/M	Rivaroxaban + Clopidogrel	Atrial Fibrillation	Y	None
Theofanopoulos et al [59]	1	T10-L3	Spontaneous	83/F	Apixaban	Atrial Fibrillation	Y	Morbidity (L anterior tibialis weakness)
Uddin et al [61]	1	C2-T2	Spontaneous	86/M	Dabigatran + Clopidogrel + ASA	Atrial Fibrillation	N	None
Kirazli et al [27]	1	T9-T10	Spontaneous	22/M	Warfarin	Mechanical Valve Replacement	Y	Morbidity mild LLE weakness
Stetkarova et al [56]	8	C5-T1	Spontaneous	59/M	Warfarin	Atrial Fibrillation	Y	None
		C1-C6	Spontaneous	68/F	Warfarin	Atrial Fibrillation	N	None
		C6-T1	Spontaneous	59/F	Warfarin	Atrial Fibrillation	N	None
		L1	Spontaneous	89/M	Warfarin	Atrial Fibrillation	N	None
		T12-L1	Spontaneous	84/F	Dabigatran	Atrial Fibrillation	N	None
		T2-T6	Spontaneous	67/F	Warfarin	Factor V Leiden Mutation	Y	Morbidity (50% of muscles less than grade 3 muscle strength)
		T11-L4	Spontaneous	68/F	Warfarin	Deep Vein Thrombosis	Y	Morbidity (50% of muscles less than grade 3 muscle strength)
		C4-C7	Spontaneous	42/M	Apixaban	Deep Vein Thrombosis + Pulmonary Embolism	Y	Morbidity (50% of muscles more than grade 3 muscle strength)
Htwe et al [22]	1	L2-L3	Spontaneous	58/M	Warfarin	Mechanical Valve Replacement	Y	Morbidity (50% of muscles less than grade 3 muscle strength) + Bowel/Bladder incontinence
Winer et al [65]	1	T6-T7	Spontaneous	73/F	Dicumarol (Warfarin)	Recurrent and progressive vertebral artery disease	Y	Paraplegia + Bowel/Bladder incontinence
Whedon et al [64]	1	C2-C4	Spontaneous Trauma	79/M	Warfarin	Atrial Fibrillation	Y	Morbidity mild BLE weakness
Ismail et al [25]	1	T11-L2	Spontaneous	72/M	Rivaroxaban + ASA	Atrial Fibrillation	Y	None
Muhammad Safian et al [49]	1	T8-T10	Spontaneous	34/M	Warfarin	Liver Failure	Y	Morbidity (50% of muscles more than grade 3 muscle strength)
Senelick et al [54]	1	L3	Spontaneous	15/F	Heparin	Ischemic Stroke	Y	None
Jacobson et al [26]	2	T2-T5	Spontaneous	61/F	AC (unspecified)		Y	Morbidity (wheelchair confinement)
		T12-L3	Spontaneous	60/F		Retinal Vein Thrombosis	Y	Morbidity bladder incontinence
		C5-C6	Spontaneous	48/M		Myocardial Infarction	Y	Morbidity L hand weakness
Ordookhanian et al [39]	1	C3-C7	Spontaneous	58/M	Warfarin	CHF	Y	UNK
Maingi et al [33]	1	T2-T5	Spontaneous	59/M	Warfarin + Dipyridamole	Thrombosis Prophylaxis	Y	Morbidity sensory deficits in BLEs
Harik et al [21]	1	T12	Spontaneous	40/M	Warfarin	Myocardial Infarction	N	None
Onobun et al [38]	1	T8-12	Spontaneous	76/M	Warfarin	Mechanical Valve Replacement	N	UNK
Truumees et al [60]	1	T7-T12	Spontaneous Trauma	72/M	Dabigatran	UNK	Y	UNK
Schicke et al [51]	1	Thoracolumbar	Spontaneous	60/M	AC (unspecified)	UNK	Y	None
Iizuka et al [24]	1	T9-T11	Spontaneous	62/F	Warfarin	Thrombophlebitis	Y	Morbidity BLE weakness and bladder dysfunction
Loya et al [32]	1	C2-C6	Spontaneous	42/M	Acenocoumarol	Deep Vein Thrombosis	Y	Morbidity L leg L5 palsy
Dahlin et al [9]	2	C3-C6	Spontaneous	49/M	Warfarin	Thrombophlebitis	Y	Morbidity (C3 paraplegic)
		T6-L1	Spontaneous Trauma	64/M	Warfarin	Mechanical Valve Replacement	Y	Morbidity (T5 paraplegic)
Byvaltsev et al [6]	1	T10-T12	Spontaneous	61/M	Warfarin	Mechanical Valve Replacement	Y	None
Morse et al [37]	1	C4-Sacrum	Post-Operative	67/M	Heparin	Post-Op Myocardial Ischemia	Y	Morbidity (back pain + bowel/bladder dysfunction)
Rahimizadeh et al [47]	1	C3-C6	Spontaneous	66/F	Rivaroxaban + ASA	Atrial Fibrillation	Y	None
Tailor et al [57]	1	T12-L1	Spontaneous	8/M	Warfarin	Mechanical Valve Replacement	N	None
Prasad et al [44]	1	T2-T3	Spontaneous	63/F	Warfarin	Pulmonary Emboli	Y	Morbidity (3/5 muscle weakness in BLEs)
Ali et al [1]	1	T11-T12	Spontaneous	65/M	Heparin	Deep Vein Thromboses	Y	None
Petrov et al [42]	1	T4-T12	Spontaneous	56/M	Acenocoumarol	Thrombophlebitis	Y	None
Alayli et al [15]	1	C2-C3, C6-C7	Spontaneous	76/F	Apixaban	Atrial Fibrillation	Y	Morbidity (weakness in all extremities and paralysis of LLE)
Hage et al [20]	1	C4-C5	Spontaneous	68/M	Warfarin	Pulmonary Embolism	Y	None
Vaya et al [62]	1	C2-C7	Spontaneous	54/F	Acenocoumarol	Mechanical Valve Replacement + Atrial Fibrillation	N	Morbidity (BLE weakness)
Tawk et al [58]	3	C2-T1	Spontaneous	68/M	Acenocoumarol	Pulmonary Embolism	Y	Morbidity (Spasticity + Bladder dysfunction)
		C3-T12	Spontaneous	49/M	Acenocoumarol	Factor 5 Leiden Mutation	Y	Morbidity (Bowel + Bladder incontinence)
		C3-C6	Spontaneous	74/M	Acenocoumarol	Ischemic CVA	Y	None
Cooper et al [8]	1	C7	Spontaneous Trauma	63/M	Warfarin	Atrial Fibrillation	Y	Morbidity (reduced fine motor skills in R hand + BLE parasthesias)oze
Sandvig et al [50]	1	L4-L5	Spontaneous	70/M	Warfarin	Atrial Flutter	Y	None
Anghelescu et al [2]	1	C3-T2	Spontaneous Trauma	80/M	Acenocoumarol	Atrial Fibrillation	Y	Morbidity (50% of muscles less than grade 3 muscle strength)
Selvaraj et al [53]	1	C6-T10	Spontaneous	30/F	Warfarin	Atrial Fibrillation	Y	Morbidity (Muscle strength 3/5 in bilateral upper and lower extremities)
El-azrak et al [16]	1	T10-T12	Spontaneous	62/F	Acenocoumarol	Atrial Fibrillation	Y	Morbidity (diffuse muscle weakness + bowel/bladder incontinence)
Sreerama et al [55]	1	L1-L5	Spontaneous	45/M	AC(unspecified)	Myocardial Infarction	Y	Morbidity (diffuse weakness + neurogenic bladder)
Ozger et al [40]	1	T11-L1	Spontaneous	53/F	Warfarin	Coronary Bypass Procedure	Y	None
Yabe et al [66]	1	C5-C7	Spontaneous	80/M	Warfarin	Atrial Fibrillation	Y	Morbidity (quadriplegia)
Goldfine et al [19]	1	C1-C7	Spontaneous	74/M	Rivaroxaban	Atrial Fibrillation	Y	UNK
Raeouf et al [46]	1	T10-L3	Spontaneous	72/F	Rivaroxaban	Atrial Fibrillation	Y	Morbidity (paraplegic)
Feret et al [18]	1	T9, L5-S2	Spontaneous	82/F	Rivaroxaban	Atrial Fibrillation	N	Morbidity (paraplegic)
Zuliani et al [69]	1	Unk	Spontaneous	86/M	Warfarin	Atrial Fibrillation	N	None
Ifuku et al [23]	1	Unk	Spontaneous	27/M	Warfarin + ASA	Mechanical Valve Replacement	Y	Morbidity (gait disturbance + dysuruia)
Lederle et al [29]	1	T6-L2	Spontaneous	69/M	Warfarin	Deep Vein Thrombosis	Y	Morbidity (Weakness in BLEs and Urinary retention)
Raasck et al [45]	1	C2-S5	Spontaneous	76/F	Warfarin	Pulmonary Embolism	N	None

Literature review of spinal epidural hematomas associated with anti-coagulation. Literature was categorized as study/author, *N*, lesion level, lesion etiology, age and gender, Anti-coagulation medication, Anti-coagulation indication, surgical decompression occurrence, and any increase in morbidity/mortality following event

### Study design and data source

A retrospective pharmacovigilance analysis was conducted utilizing data from the FDA Adverse Event Reporting System (FAERS). FAERS is a publicly accessible database that contains adverse event reports, medication error reports, and product quality complaints resulting in adverse events that were submitted to the FDA. The system relies on the voluntary, spontaneous reporting of such events by healthcare professionals, consumers, and pharmaceutical manufacturers. For this study, data were collected for SEH reports from October 1, 2003, to June 31, 2025 associated with warfarin, rivaroxaban, apixaban, and dabigatran to identify disproportionality signals and characterize patient demographics and outcomes.

Data extraction and initial processing were performed using the OpenVigil 2.1 tool. OpenVigil 2.1 is a validated pharmacovigilance instrument that interfaces with the openFDA application programming interface to query the FAERS database. The tool incorporates data cleaning algorithms, including the identification and removal of duplicate reports based on unique Individual Safety Report (ISR) codes, to enhance the quality of the retrieved data.

### Drug and adverse event definitions

The anticoagulant drugs of interest analyzed in this study were the vitamin K antagonist warfarin and the five direct oral anticoagulants (DOACs) approved in the United States: apixaban, betrixaban, dabigatran, edoxaban, and rivaroxaban.

The adverse event of interest was defined using the standardized Medical Dictionary for Regulatory Activities (MedDRA) terminology, which provides a hierarchical structure for classifying adverse event information. To ensure precision and reproducibility ISRs were queried for the specific MedDRA Preferred Term (PT) "Spinal epidural haematoma" (MedDRA code: 10,050,162) and “Spinal epidural haemorrhage”.

### Statistical analyses

Disproportionality analyses were conducted using frequentist statistical methods as calculated by the OpenVigil 2.1 tool. The validity of these embedded calculations has been previously described and established [5, 43]. The principle of disproportionality analysis is to assess whether a specific adverse event is reported more frequently in association with a particular drug compared to its reporting frequency with all other drugs in the database. For the analysis of each anticoagulant of interest, the comparator group consisted of all other drugs reported in the FAERS database during the specified study period.

The association between each drug and the adverse event was quantified using a standard 2 × 2 contingency table:

**Table:** 

	Reports of Spinal Epidural Hematoma	Other Adverse Events
Drug of Interest	A	B
All other Drugs	C	D

Three primary measures of disproportionality were calculated, each with a 95% Confidence Interval (CI):

1. Reporting Odds Ratio (ROR): The odds of a report of SEH occurring with the drug of interest compared to the odds of the event occurring with all other drugs in the database.
  $$ROR= \frac{A*D}{B*C}$$
2. Proportional Reporting Ratio (PRR): The proportion of reports for a given drug that are linked to SEH, divided by the corresponding proportion for all other drugs combined.
  $$PRR=\frac{A/(A+B)}{C/(C+D)}$$
3. Relative Reporting Ratio (RRR): The ratio of the observed number of reports of SEH with the drug of interest to the number of expected reports, under the null hypothesis of no association between the drug and the event.
  $$RRR= \frac{Observed}{Expected}= \frac{A*(A+B+C+D)}{\left(A+B\right)*(A+C)}$$

To enhance the specificity of signal detection and minimize false-positive results inherent in spontaneous reporting data, a potential safety signal for a "likely true adverse event" was defined by the fulfillment of three pre-specified criteria, based on the established methodology of Evans et al. [17].

1. A total of more than three reports for the drug-event combination (A > 3).
2. A chi-squared test value with Yates' correction greater than 4.
3. A Proportional Reporting Ratio greater than 2 (PRR > 2).

### Data availability

Data derived from public domain resources. The data that support the findings of this study are available in the openFDA portal at https://open.fda.gov/ and OpenVigil 2.1 at http://openvigil.sourceforge.net/. These data were derived from the following resources available in the public domain: FDA Adverse Event Reporting System (FAERS).

## Results

According to our literature search, the most common lesion location was the cervical spine. The male to female ratio was 44:29 and the average age was 64 years old. The most common anti-coagulation medication was warfarin for a most common indication of atrial fibrillation. Warfarin being the most common concurrent anti-coagulation medication used during the presence of a spinal epidural hematoma further substantiates the objective data derived from the FAERS database. Furthermore, the subsequent increase in morbidity and mortality related to the severity of the spinal epidural hematoma overscores the seriousness of the issue. While this data is not causal, it highlights the importance of this systematic review.

During the study period from Q4/2003 to Q2/2025, a total of 143 reports of spinal epidural hematoma were identified for the anticoagulants of interest. Warfarin was associated with the highest number of reports (*n* = 83), followed by rivaroxaban (*n* = 28), apixaban (*n* = 25), and dabigatran (*n* = 7). Betrixaban and edoxaban were excluded from further analysis due to a minimal number of reports (0 and 1, respectively).

**Fig. 1:**
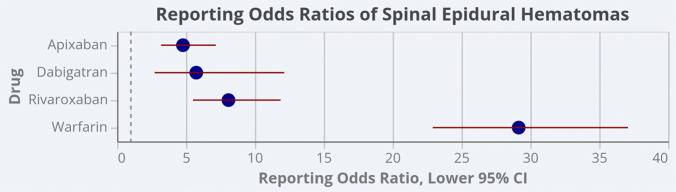
Reporting odds ratios of spinal epidural hematoma

All four analyzed drugs (warfarin, rivaroxaban, dabigatran, and apixaban) met the pre-specified criteria for a likely true adverse event signal (*n* > 3, Chi-Squared with Yates’ correction > 4, and PRR > 2). Warfarin demonstrated the strongest signal of disproportionality, with a ROR of 29.2 (95% CI 22.9–37.1), PRR of 29.1 (95% CI 22.9–37.1), and a chi-squared value of 1777.99. Among the DOACs, rivaroxaban showed the highest disproportionality with an ROR of 8.09 (95% CI 5.51–11.9), followed by dabigatran with an ROR of 5.75 (95% CI 2.72–12.1), and apixaban with an ROR of 4.78 (95% CI 3.19–7.17). The complete statistical analyses are presented in Table 2, and a visual comparison of the RORs is provided in Fig. 1. Demographic and outcome data are summarized in Supplemental Table 3. The mean age of patients with SEH reports was notably lower for Warfarin (62 years) compared to the DOACs, where the mean age was 75–76 years. Serious outcomes were reported for all drugs. For Warfarin, 9 deaths and 66 cases of hospitalization or disability were reported. For Rivaroxaban, 8 deaths and 15 hospitalizations or disabilities were reported. No deaths were reported for Dabigatran or Apixaban, though cases of hospitalization or disability were noted (5 and 21, respectively).

**Table 2:** Statistical analyses of spinal epidural hematoma/hemorrhage with Warfarin and DOACs

Drug	Rate (%)	Chi-Squared with Yates' correction	Drug Spinal EDH Reports	Other Drugs Spinal EDH Reports	Drug All Other Adverse Events	Other Drugs All Other Adverse Events	RRR (95% CI)	PRR (95% CI)	ROR (95% CI)	Adverse Event?*
Warfarin	0.07	1777.99	83	329	118,487	13,691,300	23.5 (18.5–29.7)	29.1 (22.9–37.1)	29.2 (22.9–37.1)	YES
Rivaroxaban	0.02	138.495	28	384	123,419	13,686,400	7.6 (5.18–11.1)	8.08 (5.51–11.9)	8.09 (5.51–11.9)	YES
Dabigatran	0.02	22.506	7	405	41,400	13,768,400	5.67 (2.69–12)	5.75 (2.72–12.1)	5.75 (2.72–12.1)	YES
Apixaban	0.01	66.703	25	387	184,022	13,625,800	4.55 (3.04–6.82)	4.78 (3.19–7.17)	4.78 (3.19–7.17)	YES

Statistical analyses of spinal epidural hematomas from relevant reporting data retrieved on September 10, 2025 covering from Q4/2003 to Q2/2025. Anti-coagulants Betrixaban and Edoxaban were excluded due to minimal reports (0 and 1 respectively)

RRR = Relative Reporting Ratio

PRR = Proportional Reporting Ratio

ROR = Reporting Odds Ratio

Rate (%) calculated as (number of spinal epidural hematoma reports for the drug/total adverse event reports for the drug) × 100

* Adverse assessed according to the criteria of Evans 2001 (*n* > 3, Chi-Squared with Yates’ correction > 4, PRR > 2)

The reported indications for anticoagulant use are detailed in Table 4. Atrial fibrillation was the most frequently cited indication for all four drugs. Warfarin also had a significant number of reports for pulmonary embolism and deep vein thrombosis. A substantial portion of reports for all drugs had an unknown indication, accounting for over 50% of the cases for warfarin.

## Discussion

The incidence of SEH overall is relatively rare, with the overall pooled incidence of post-operative SEH from a 2022 meta-analysis of 40 studies being 0.52% [7]. Despite this, it is an incredibly dangerous complication with risk of significant morbidity and mortality, with possible adverse outcomes including paraplegia, quadriplegia, and even death [68]. In this study, reports of SEH incidence with certain anticoagulants were disproportionally high with warfarin demonstrating the strongest signal of the four drugs analyzed. The comprehensive nature of this study allows us to have confidence in our results seeing as warfarin, rivaroxaban, apixaban, and dabigatran were all included in the analysis, and betrixaban and edoxaban were excluded due to not meeting the methodological constraint delineated by Evans et al. [17] Furthermore, demographic data demonstrated that normal aging had no influence on the outcomes of the study seeing that the mean age of warfarin recipients was substantially younger (62 years) compared to DOAC recipients (75–76 years), and warfarin still had higher reports of hospitalization (66) when compared to Factor Xa inhibitors (15 & 21) and the direct thrombin inhibitor (5). Additionally, patient gender and anticoagulation indication had no bearing on the study as they were evenly distributed throughout. Our data emphasizes the legitimacy of the association between warfarin and rivaroxaban having increased disproportionality signals. While this study utilizes a large, national database, several important limitations must be noted. First, the FAERS database relies on voluntary submission from healthcare professionals, consumers, and manufacturers, meaning the enumeration of adverse events is far from comprehensive. Comparing disproportionality between different agents, such as warfarin and DOACs, can be inherently misleading due to variability in sampling and overall drug utilization. The size of utilization directly impacts these metrics; a drug prescribed more frequently may have a larger raw number of reports, and differences in market longevity between warfarin and DOACs introduce significant timeline biases. Furthermore, disproportionality analysis is susceptible to reporting bias. Severe or well-known complications, like SEH in patients on warfarin, may be disproportionately reported compared to lesser-known associations or minor adverse events. Therefore, the differences in signal strength cannot confidently establish absolute risk or definitive causality, and the results should strictly be interpreted as a signal-generating analysis.

There is no significantly established relationship between the incidence of SEH and anticoagulant use. In fact, there are conflicting findings with the same 2022 meta-analysis stating there is no significant variation in the incidence of SEH between patients who received perioperative anticoagulation therapy, and a few different small case series and individual case reports showing that patients on anticoagulation therapy had spinal epidural hematomas [7, 30, 34, 36, 43]. It is understandable that clinicians and scientists alike are eager to find explanations to help elucidate why their patients developed a relatively rare but dangerous pathology like SEH, but the data available constitutes a paucity at best. While the relationship between SEH, and anticoagulant therapy is mechanistically explicable, with these drugs reducing the rate of clots but conversely increasing the risk of hemorrhage, there exists insufficient evidence in the existing literature to make data driven decisions. With that being said, this study helps pinpoint drugs of interest with its disproportionality analysis.

Starting with warfarin, this study showed that it demonstrated the strongest signal of disproportionality, with a ROR of 29.2 (95% CI 22.9–37.1), PRR of 29.1 (95% CI 22.9–37.1), and a chi-squared value of 1777.99. The ROR finding means that of all the reported adverse events for warfarin, randomly picking one is 29.2 times more likely to be SEH than if an adverse event was randomly picked for any other drug. The PRR finding means that the proportion of Warfarin event reports that are SEH is 29.1 times higher than the proportion of SEH reports for all other drugs combined. The significance of the ROR and PRR values being similar confirms that SEH is a rare event, as in common findings these values can differ substantially. Lastly, the high chi-squared value just shows that it is incredibly unlikely that these findings occurred by chance as the observed value dwarfs the expected value. The existing literature has limited data characterizing the incidence of SEH in patients on warfarin with several case reports and a few small single institution patient series (*N* < 20) indicating that these cases have occurred but no large analysis has been conducted to determine a incidental or causal relationship [17, 30, 41]. It is easy to logically connect the pharmacologic mechanism of warfarin with an increased risk of SEH, as it is a vitamin K epoxide reductase inhibitor, which means it increased the risk of bleeding by impairing the functioning of the extrinsic coagulation cascade through the depletion of factors II, VII, IX, and X, leaving patients more prone to bleeding [11].

Of the remaining drugs, which were all DOACs, rivaroxaban showed the highest disproportionality with an ROR of 8.09 (95% CI 5.51–11.9), PRR of 8.08 (95% CI 5.51–11.9), and chi-squared value of 138.50. This was followed by dabigatran with an ROR of 5.75 (95% CI 2.72–12.1), PRR of 5.75 (95% CI 2.72–12.1), and chi-squared Value of 22.51. Lastly, apixaban had an ROR of 4.78 (95% CI 3.19–7.17), PRR of 4.68 (95% CI 3.19–7.17), and chi-squared value of 66.70. The significance of these values can be interpreted in the same way that the values for warfarin could be, and they also show that there is strong signal of a relationship between these 3 DOACs and SEH, however, not as strong of a signal as warfarin. Like in the case of warfarin, it is not hard to draw a mechanistic connection between the actions of these drugs and the potential for increased risk of SEH, as they are all anticoagulants, with rivaroxaban and apixaban being Factor Xa inhibitors and dabigatran being a direct thrombin inhibitor (as compared to an indirect thrombin inhibitor like heparin). As their names suggest, these drugs inhibit factor Xa and thrombin of the coagulation cascade respectively, leaving the patient at more risk of bleeding [10, 12–14]. Despite this seemingly obvious mechanistic relationship, these is a paucity of evidence of this relationship, with the existing literature being limited to case reports of incidences involving patients developing SEH while on these drugs [4, 28, 31, 34, 35, 52, 59, 61].

Our data indicates a clear association between warfarin and SEH. This is largely well accepted in the community, and a calculated risk most clinicians are required to take. DOACs are largely regarded similarly but have been shown to be safer in terms of overall bleeding risk. In one study, there was a significantly lower associated risk of major bleeding with DOACs when comparted to warfarin [67]. Another study showed DOACs have an overall decreased risk in intracranial hemorrhage when compared to warfarin [3]. There are a multitude of theories hypothesizing why DOACs are safer than warfarin, including that warfarin targets the entire vitamin K cascade as opposed to a single target [63]. Nevertheless, our study hopes to further that ideology and attempts to delineate any difference within the DOAC class. We found rivaroxaban to have an appreciably increased disproportionality signal when compared to all other DOACs. In addition, in regard to mortality there were 9 reported deaths using warfarin and 8 using rivaroxaban, with none for dabigatran and apixaban. Finally, our literature review of SEH portrays that warfarin was the most used anti-coagulation followed by rivaroxaban. This analysis identified a disproportionately high reporting signal for SEH with Warfarin and Rivaroxaban compared to other anticoagulants. While these findings do not establish causality or absolute risk, they suggest a need for heightened vigilance for these medications when anticoagulation is required. Further research is needed to elucidate if there is a definitive relationship between the use of anticoagulant medication and incidence of SEH as this disproportionality analysis can only indicate signal but cannot draw conclusions on incidence or causality, and the existing literature on this topic is limited to case reports reflecting clinicians’ intuition of said relationship.

## Limitations

To expand, this study has several important limitations inherent to its reliance on a spontaneous reporting system like FAERS. First and foremost, disproportionality analysis can identify a signal but cannot establish causality or determine the incidence of an adverse event, largely because FAERS lacks denominator data. The number of reports is influenced by many factors, including the length of time a drug has been on the market, differences in overall prescribing volume, media attention, and the severity of the event, a phenomenon known as reporting bias. The much stronger signal for warfarin could be partially attributable to its longer market presence, higher utilization size over time, and greater notoriety for causing bleeding complications.

Second, the data within FAERS reports are often incomplete. Critical clinical information, such as the INR for patients on warfarin, concomitant use of other medications (e.g., antiplatelets), or the presence of other risk factors for SEH like recent spinal procedures or trauma, is frequently missing. The high percentage of reports with an "unknown" indication for anticoagulation underscores this limitation.

Third, while the OpenVigil tool employs algorithms to remove duplicate reports, some may persist, potentially inflating the number of cases. Finally, this analysis reflects reported associations and should be interpreted as a signal-generating study. Definitive conclusions about the comparative risk of SEH between these agents would require large-scale, prospective, controlled studies.

## Conclusion

Despite the limitations of the analysis methodology and database of study, pharmacovigilance disproportionality analyses remain an essential tool for detecting rare but serious adverse events that may not be apparent in pre-marketing trials, and our findings warrant continued vigilance for SEH across these commonly used anticoagulants. Additionally, it is worth noting that our findings most likely reflect the intuition of risk that many clinicians and scientists expected, which we can gather from the myriads of case reports attempting to provide scientific accounts of a potential relationship, however, our study is the first to quantitatively elucidate a potential signal of risk between SEH and anticoagulant use. Despite the strong signal of this study, further research is needed to delineate the relationship between SEH and these anticoagulants.

## Supplementary Information

Below is the link to the electronic supplementary material.

ESM 1 Supplementary material 1 (DOCX 14.7 KB)

ESM 2 Supplementary material 2 (DOCX 14.8 KB)

## Data Availability

Data derived from public domain resources The data that support the findings of this study are available in the openFDA portal at https://open.fda.gov/ and OpenVigil 2.1 at http://openvigil.sourceforge.net/. These data were derived from the following resources available in the public domain: FDA Adverse Event Reporting System (FAERS).
