# Supplementary material for: Pharmacovigilance study of spinal epidural hematoma reports associated with direct oral anticoagulants and warfarin
Source: Acta Neurochir (Wien). 2026 Apr 30;168(1):146. doi: 10.1007/s00701-026-06860-0 (PMC13287272; doi:10.1007/s00701-026-06860-0)
Supplement: Supplementary file 1 — Supplementary material 1 (DOCX 14.7 KB) [file 701_2026_6860_MOESM1_ESM.docx]

**Supplemental Table 3**. Reported Demographic and Outcome Data

| Drug | Age, Mean (SD) | Male, n (%) | Female, n (%) | Deaths, n (%) | Hospitalization or Disability, n (%)* |
| --- | --- | --- | --- | --- | --- |
| Warfarin | 64.5 (16.1) | 33 (39.8%) | 41 (49.4%) | 9 (10.8%) | 66 (79.5%) |
| Rivaroxaban | 76.1 (11.2) | 12 (42.9%) | 14 (50.0%) | 8 (28.6%) | 15 (53.6%) |
| Dabigatran | 76.3 (6.2) | 4 (57.1%) | 3 (42.9%) | 0 (0.0%) | 5 (71.4%) |
| Apixaban | 75.0 (5.8) | 11 (44.0%) | 13 (52.0%) | 0 (0.0%) | 21 (84.0%) |

**Supplemental Table 3 Caption**: Demographic and outcome data for patients receiving anti-coagulation medication. Data was stratified by specific medication utilized.

* Sex unknown for 9 patients taking Warfarin, and 2 taking Rivaroxaban
