# Supplementary material for: Pharmacovigilance study of spinal epidural hematoma reports associated with direct oral anticoagulants and warfarin
Source: Acta Neurochir (Wien). 2026 Apr 30;168(1):146. doi: 10.1007/s00701-026-06860-0 (PMC13287272; doi:10.1007/s00701-026-06860-0)
Supplement: Supplementary file 2 — Supplementary material 2 (DOCX 14.8 KB) [file 701_2026_6860_MOESM2_ESM.docx]

**Supplemental Table 4**. Reported Drug Indications

| Indication | Warfarin | Rivaroxaban | Dabigatran | Apixaban |
| --- | --- | --- | --- | --- |
| Atrial Fibrillation | 15 (18.1%) | 9 (32.1%) | 4 (57.1%) | 6 (24.0%) |
| Pulmonary Embolism | 9 (10.8%) | 0 (0.0%) | 0 (0.0%) | 0 (0.0%) |
| DVT | 7 (8.4%) | 1 (3.6%) | 0 (0.0%) | 0 (0.0%) |
| Cardiac Disease | 6 (7.2%) | 1 (3.6%) | 0 (0.0%) | 2 (8.0%) |
| CVA/Thrombus Prophylaxis | 2 (2.4%) | 3 (10.7%) | 0 (0.0%) | 6 (24.0%) |
| Unknown | 44 (53.0%) | 14 (50.0%) | 3 (42.9%) | 11 (44.0%) |
| **Total** | **83** | **28** | **7** | **25** |

**Table 4 Caption:** Medical indication for anti-coagulation utilization. Anti-coagulation medication is stratified by each indication.
